# Experimental study on bed pressure around geotextile mattress with sloping plate

**DOI:** 10.1371/journal.pone.0211312

**Published:** 2019-01-25

**Authors:** Liquan Xie, Yehui Zhu, YanHong Li, Tsung-Chow Su

**Affiliations:** 1 College of Civil Engineering, Tongji University, Shanghai, China; 2 School of Naval Architecture, Ocean and Civil Engineering, Shanghai Jiaotong University, Shanghai, China; 3 Department of Ocean and Mechanical Engineering, Florida Atlantic University, Boca Raton, Florida, United States of America; China University of Mining and Technology, CHINA

## Abstract

A geotextile mattress with sloping curtain is a newly proposed countermeasure against river and estuarine scour. In previous laboratory experiments, a geotextile mattress with sloping curtain was capable of protecting the bed downstream from scour and stimulating sediment deposition on both sides. However, the seepage scour under its geotextile mattress is inadequately researched at present. In this study, the Geotextile Mattress with Sloping Plate (GMSP) is proposed based on the simplification of the geotextile mattress with sloping curtain with the construction feasibility considered. A series of experiments was conducted to investigate the pressure distribution around the GMSP and the averaged seepage hydraulic gradient beneath its mattress. The results indicate remarkable pressure difference on two sides of the GMSP. The minimum bed pressure appears about 1.3 times the plate height downstream to the GMSP. The averaged seepage hydraulic gradient beneath the mattress increases with the sloping angle increasing from 35° to 60° in general. The averaged hydraulic gradient also ascends as the relative plate height increases, but reduces as the opening ratio increases at opening ratios greater than 0.143. The safety boundary for the averaged hydraulic gradient under the geotextile mattress of the GMSP could get much smaller than the critical hydraulic gradient of piping and can easily be overwhelmed. This phenomenon can mainly be attributed to the discontinuous contact between the mattress and the seabed. A suggestion for the parametric design of the GMSP is to extend the width of the mattress to reduce the risk of seepage failure.

## Introduction

**Fig 1:**
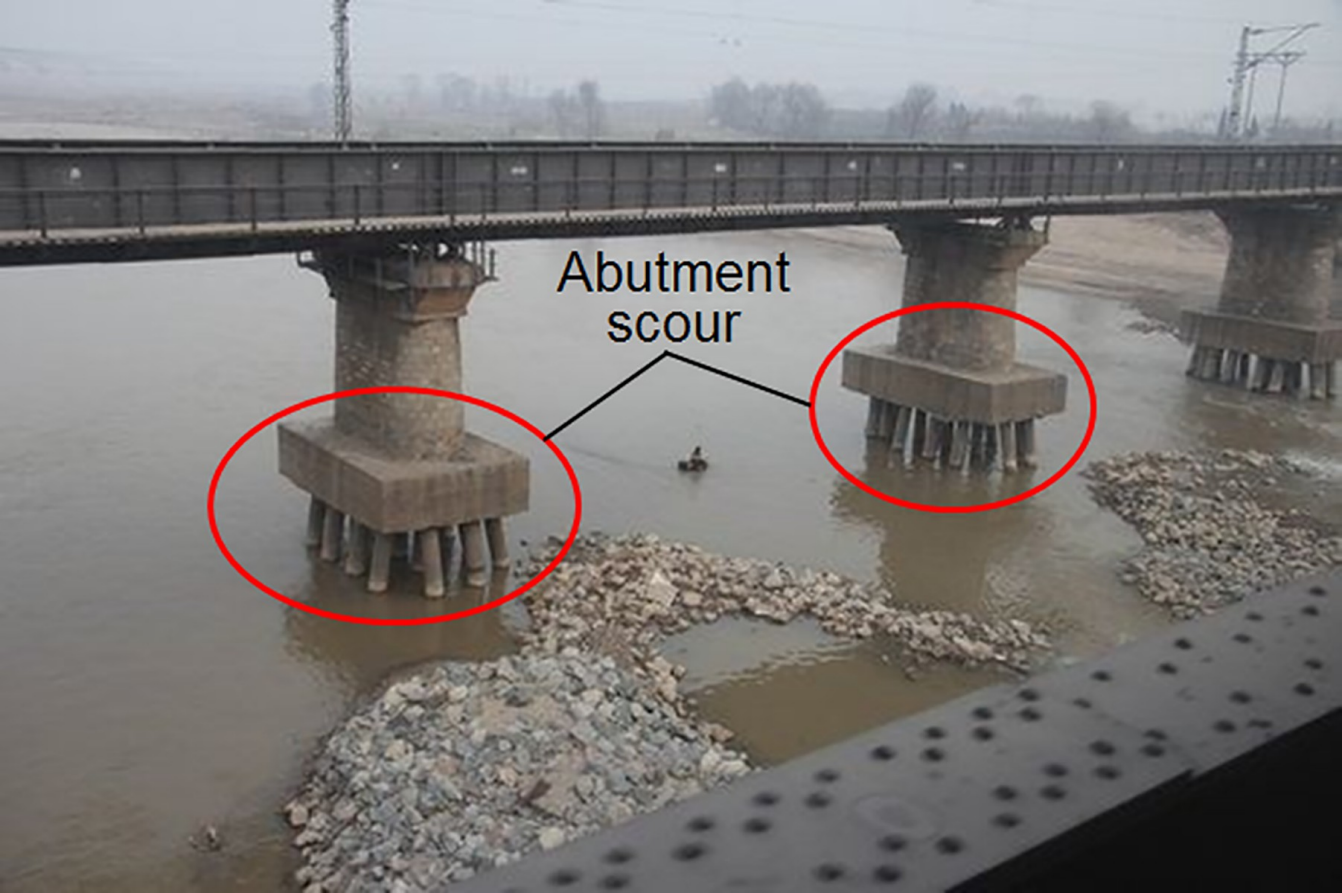
Erosion at the piers of Longhai Railway bridge in Xianyang, China (Modified from the photo in China Construction News [22]).

**Fig 2:**
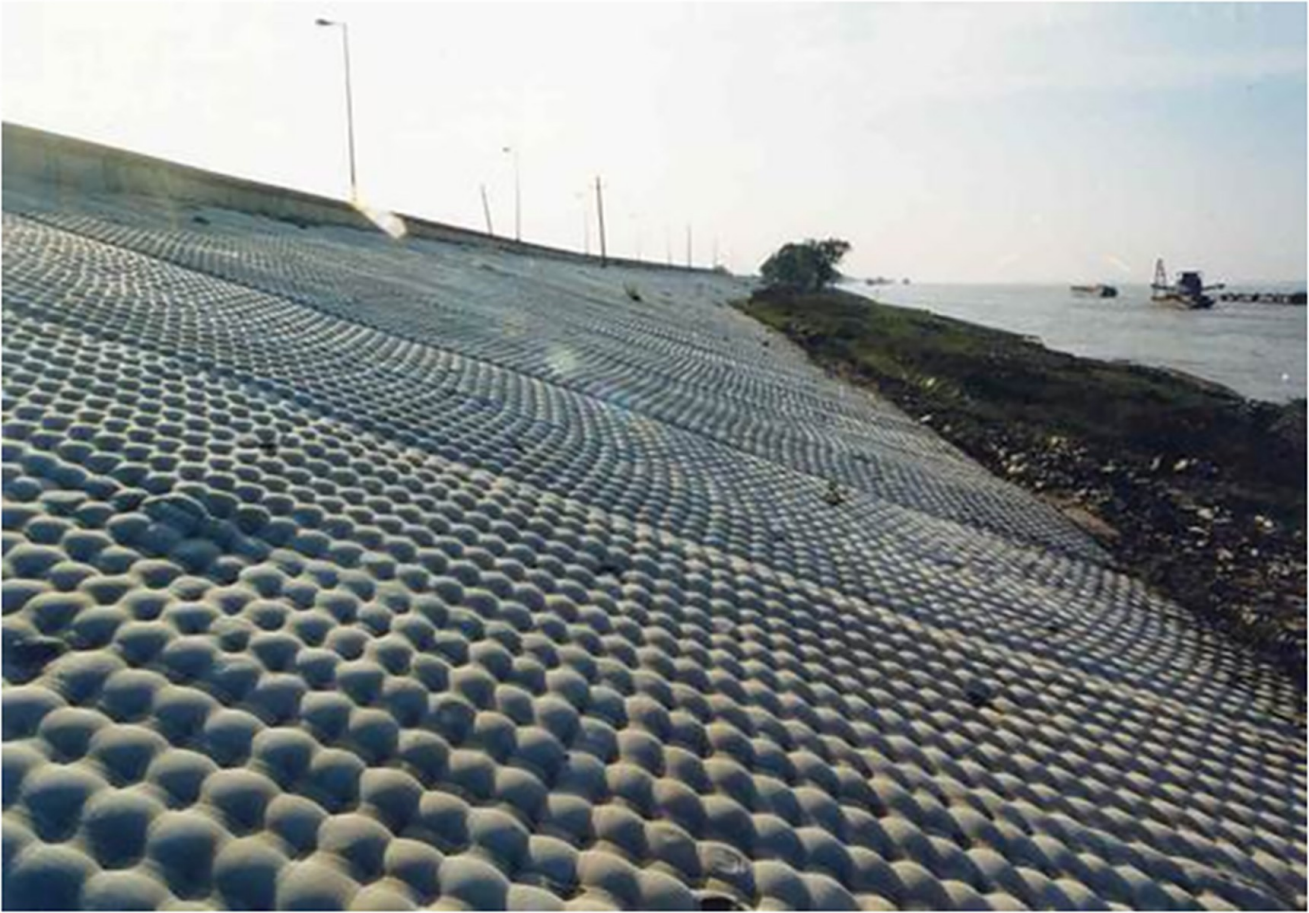
Revetment made of geosynthetic structures on the bank of the Yangtze River in Jiujiang, China.

Scours in river channels and coastal areas are long-lasting threats to the underwater structures and dikes. The failure of these structures and dikes could lead to considerable economic losses and heavy casualties. The scour on the river banks could shape the bank slope much steeper, which would cause bank collapses [1, 2]. Underwater scours may also endanger the structures installed on the seabed like bridge piers [3, 4], pipelines [5–7] and wind turbine foundations [8–10]. For example, degradation of river channel and scour around bridge piers may cause the exposure of the pier foundations, including the footings and the piles (see Fig 1). The exposure of bridge foundations will surely endanger the stability of bridges and may result in bridge failure and thus trigger local traffic paralysis and great economic losses. The present measures against the scour can be mainly divided into two sections according to their principles: enhancing the stability of the erodible bed and modifying the flow structure. The former mainly includes toe protection [11], revetments [12, 13] and mattresses [14], while the latter includes groins [15, 16] and submerged breakwaters [17, 18]. However, many of the measures consolidating the stability of sediment on river bed and banks, like the revetments and mattresses, would lead to some environmental and ecological problems. These structures were reported to affect the abundance of fish species in local reaches [19] probably because they completely cover the bank slopes and river beds and cut off the interaction between the channel flow and the sediment on banks and river beds [20]. In China, a large quantity of revetments and mattresses with a full coverage of river banks or river beds scatter along the middle and downstream reaches of the Yangtze River (see Fig 2), bringing about satisfying protection effects and great impacts on local ecological systems as well. At the same time, measures attempting to change the flow patterns cost heavily in construction [21] and maintenance. Therefore, some improvements have been developed based on these adverse effects.

**Fig 3:**
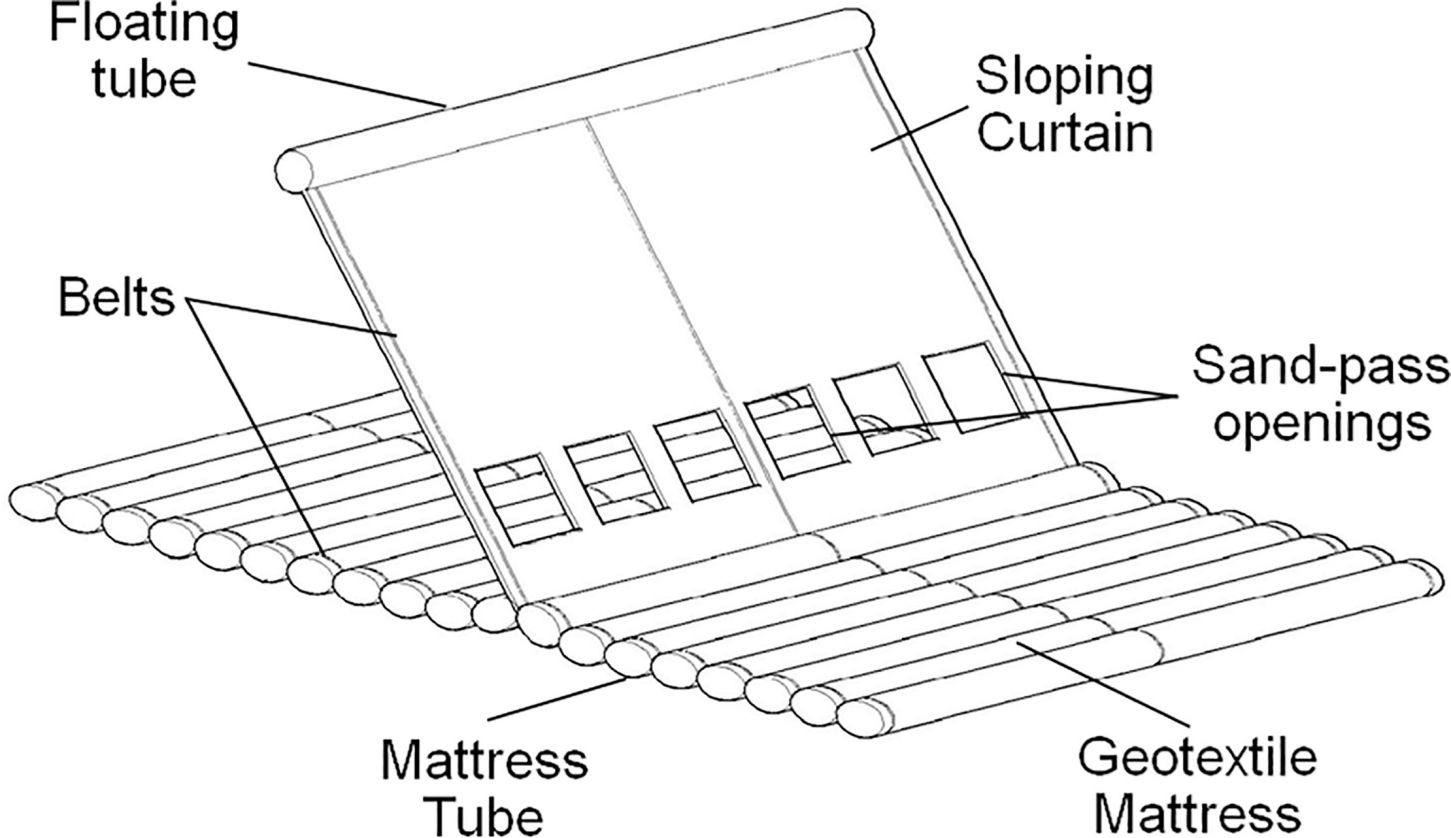
Sketch of Geotextile mattress with sloping curtain (GMSC).

A geotextile mattress with sloping curtain (GMSC) is a newly proposed countermeasure against the scour around the underwater structures first introduced in a former paper of the authors [23]. A GMSC contains two main parts: a geotextile mattress and a sloping curtain (see Fig 3). The geotextile mattress is constituted of a row of mattress tubes made of geotextile fabric. The tubes are and filled with sand, gravel, and sometimes dredged materials. The gravity of the mattress provides the stability of the entire structure. The sloping curtain which is made of two sheets of geotextile fabric is sewn on the mattress on the bottom side and attached to the floating tube on the top edge. When the GMSC is placed in still water, the floating tube pulls the curtain straight up. When the GMSC faces a steady flow, the current pushes the curtain forward and the curtain inclines to the downstream side. The curtain is thus termed as a “sloping” curtain. Some sand-pass openings are set near the bottom of the curtain for the passage of near-bottom sediment load. The GMSC is equipped with some belts to improve the intensity of the structure. The GMSC can be deployed along the bank slopes or river beds separately, without covering the interface of river flow and banks (or river beds) completely. Scour and erosion can thus be controlled with the local ecological system preserved.

**Fig 4:**
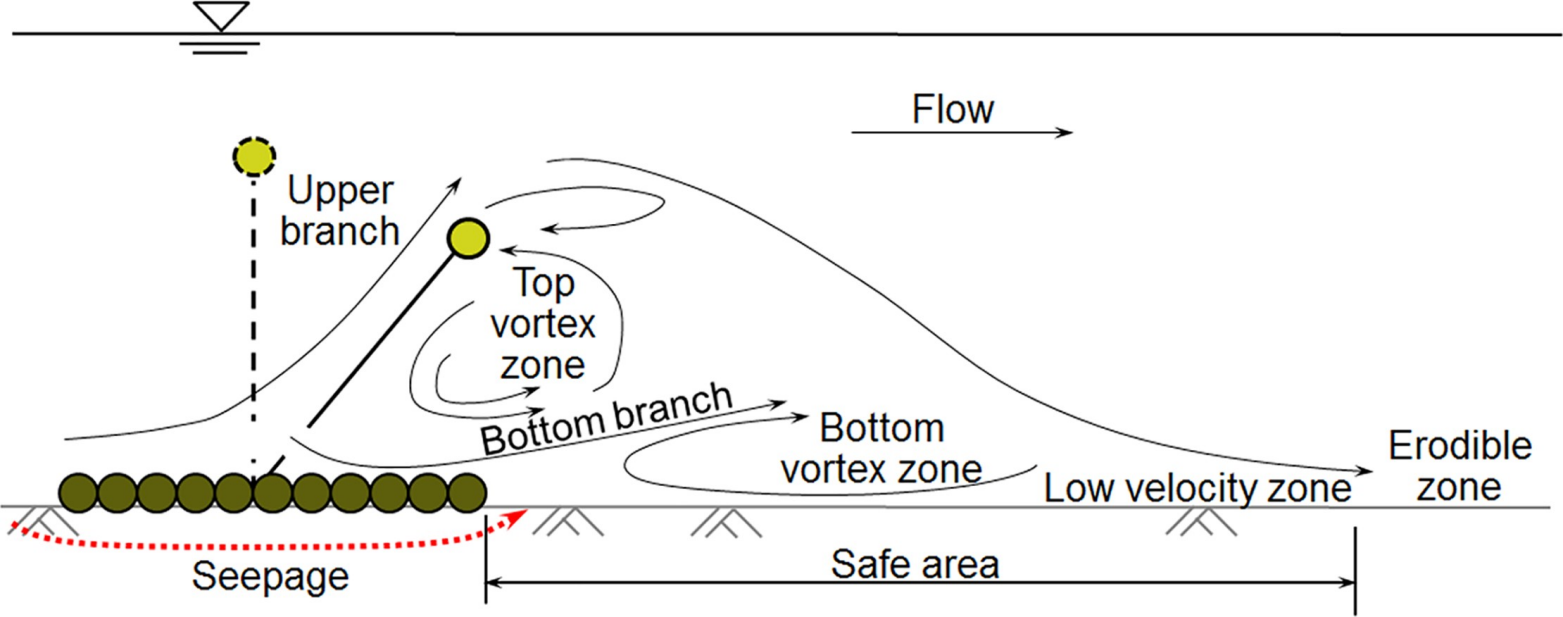
Flow structure around and seepage beneath the GMSC.

Xie et al. [24] revealed the elementary working mechanisms of a GMSC structure (see Fig 4). In a steady current, a GMSC separates the incoming flow into two branches: the upper branch and the bottom branch. The upper branch clings to the sloping curtain and climbs over the floating tube. The bottom branch with a high fraction of bed load rushes through the sand-pass openings. The blockage effect of the GMSC creates a series of lee wake vortices in two vortex zones: the top zone and the bottom zone. The top vortex zone locates far above the bed and its effect on the bed can be neglected. The bottom vortex helps to create a long low velocity zone on the leeside of the GMSC. The bottom vortex and the low velocity zone are actually the safe area from scour. The sediment load passing through the opening with the bottom flow branch will deposit in this area, forming a sand dune. The GMSC should be deployed so that the structure or bank to be protected falls inside the safe zone of the GMSC, where the flow velocity is lower. The range of the safe zone is affected by various factors, including the parameters of GMSC, the flow parameters, the properties of the bed boundary, etc. In practice, the range of the safe zone is mainly determined with laboratory experiments and numerical simulations.

Some studies on the effectiveness and features of the GMSC have been conducted. Xie and Liu [23] conducted a series of experiments and numerical simulations to verify the performance of the GMSC in the scour control on sand beds. The GMSC was proved to be capable of reducing the bed shear stress in a long section downstream and providing a safe zone up to 21 times of the GMSC curtain height on the leeside of the curtain. Xie et al. [24] studied the sediment deposition on the downstream side of the GMSC in live bed condition experimentally. Sand dunes were found at both upstream and downstream sides of the GMSC and the sediment profile on the leeside of the GMSC showed a close relation with the opening ratio of the GMSC.

Li and Yu [25] carried out an experimental study on the size of the bottom vortex and the location of reattachment point on the leeside of the sloping curtain using a simplified Particle Image Velocimetry (PIV) system. The experiment results showed that the characteristics of the bottom vortex are dominated by the size of the curtain and sand-pass openings. Xie et al. [26] conducted a series of 3D numerical calculations to study the flow structure on the leeside of the GMSC. The results showed that the water surface variation is more significant when the GMSC is not equipped with sand-pass openings. Gu et al. [27] investigated the effects of openings on the length of the protected zones through numerical simulations. The results indicated that the length of protection zone is almost independent of the opening ratio and the GMSC without openings offers a longer protected zone but is short of sediments deposition.

Xie et al. [28] studied the response of bed pressure distribution adjacent to a GMSC in wave conditions with a hydraulic flume. The result indicated that the largest cyclic force appears at about a distance of the curtain height to the centerline of the GMSC.

**Fig 5:**
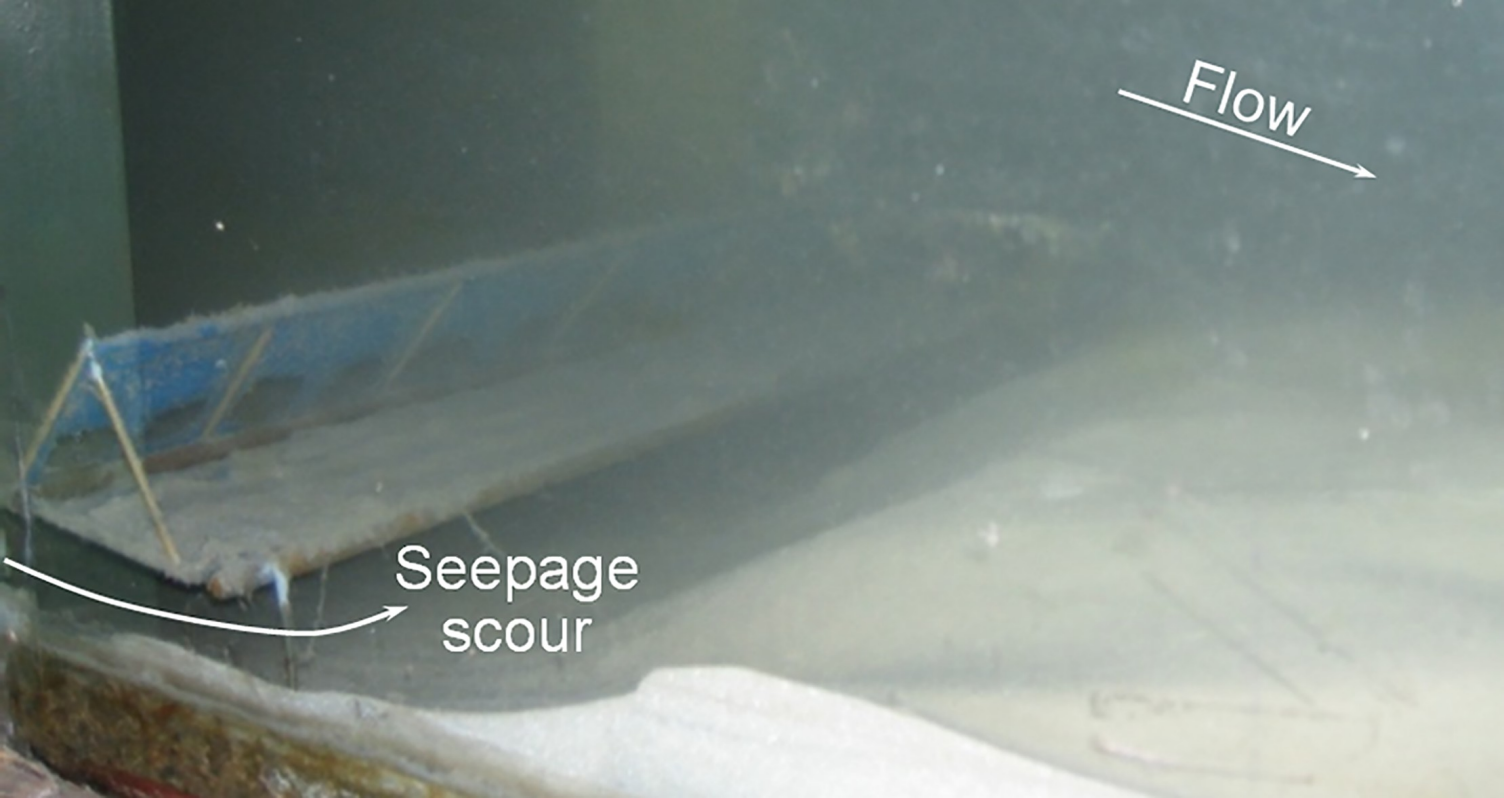
Seepage scour under the GMSC after 38 hours of flow by Xie et al. [24].

However, in the live-bed experiments by Xie et al. [24], a scour hole appeared beneath the geotextile mattress after the 38 hours of flow (see Fig 5), indicating that the discussion on the stability and safety of the GMSC can be inadequate. The excessive seepage flow induced by the pressure difference on two sides of the GMSC is among the leading causes of the scour underneath the geotextile mattress [23]. When the seepage hydraulic gradient is over a critical value, piping will occur below the mattress. Thus the GMSC will finally be pushed forward or rolled up by the current, leading to the failure of the GMSC structure.

In this study, the Geotextile Mattress with Sloping Plate (GMSP) is proposed based on the simplification of the GMSC with the construction feasibility considered. A series of tests were designed to measure the bed pressure distribution pattern around the GMSP. The effects of three design parameters on pressure difference on two sides of the GMSP and seepage hydraulic gradient under the geotextile mattress was evaluated. The overall aim of this study is to offer some preliminary reference to the design of GMSP based on the effects of geometry parameters on the seepage stability of GMSP.

## Geotextile mattress with sloping plate

**Fig 6:**
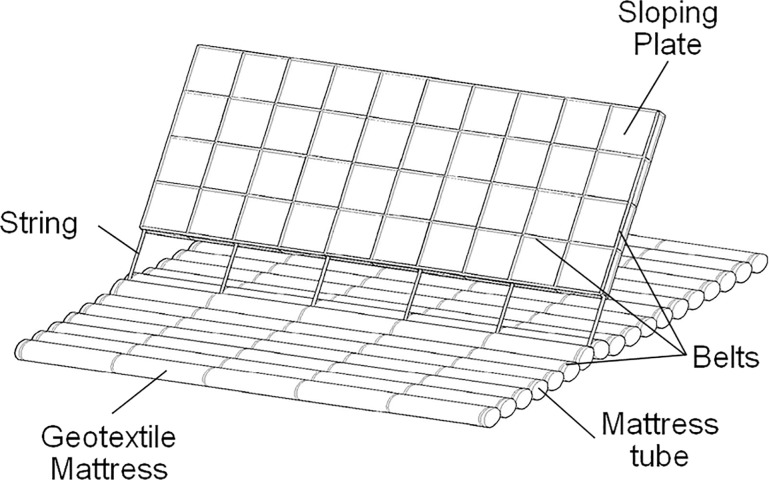
Sketch of geotextile mattress with sloping plate (GMSP).

A series of simplifications are proposed on the structure of the GMSC to make it friendlier in the engineering construction (see Fig 6). The sloping curtain is replaced with a floating plate which will also lean to the leeside when placed in steady current. Thus the improved device is termed as the Geotextile Mattress with Sloping Plate (GMSP). The sloping plate can be made of floating materials like foam polymers or inflatable structures. In practical engineering, the dimensions and the material of the sloping plate are designed based on various factors, including the size and properties of the bed or structures to be protected, the flow parameters like flow depth and flow velocity. The floating tube on the top of the curtain is removed. The sloping plate is anchored to the geotextile mattress with a series of strings on the bottom edge. The gap between the bottom edge of the sloping plate and the mattress serves as the sand-pass openings. The belts are used on the sloping plate for strengthening the integrality. The other components of the GMSC remain the same.

The changes on the structures can improve the convenience in engineering construction and maintenance. In the deployment phase, the geotextile mattress and the floating component of a GMSP can be installed separately, which avoids the potential controlling troubles due to the buoyancy force provided by the floating tube of the GMSC and the installation can be more accurate. In the maintenance afterwards, the sloping plate can be replaced conveniently and separately, and there is no need of replacing the mattress at the same time like the GMSC, thus saving plenty of time and cost.

**Fig 7:**
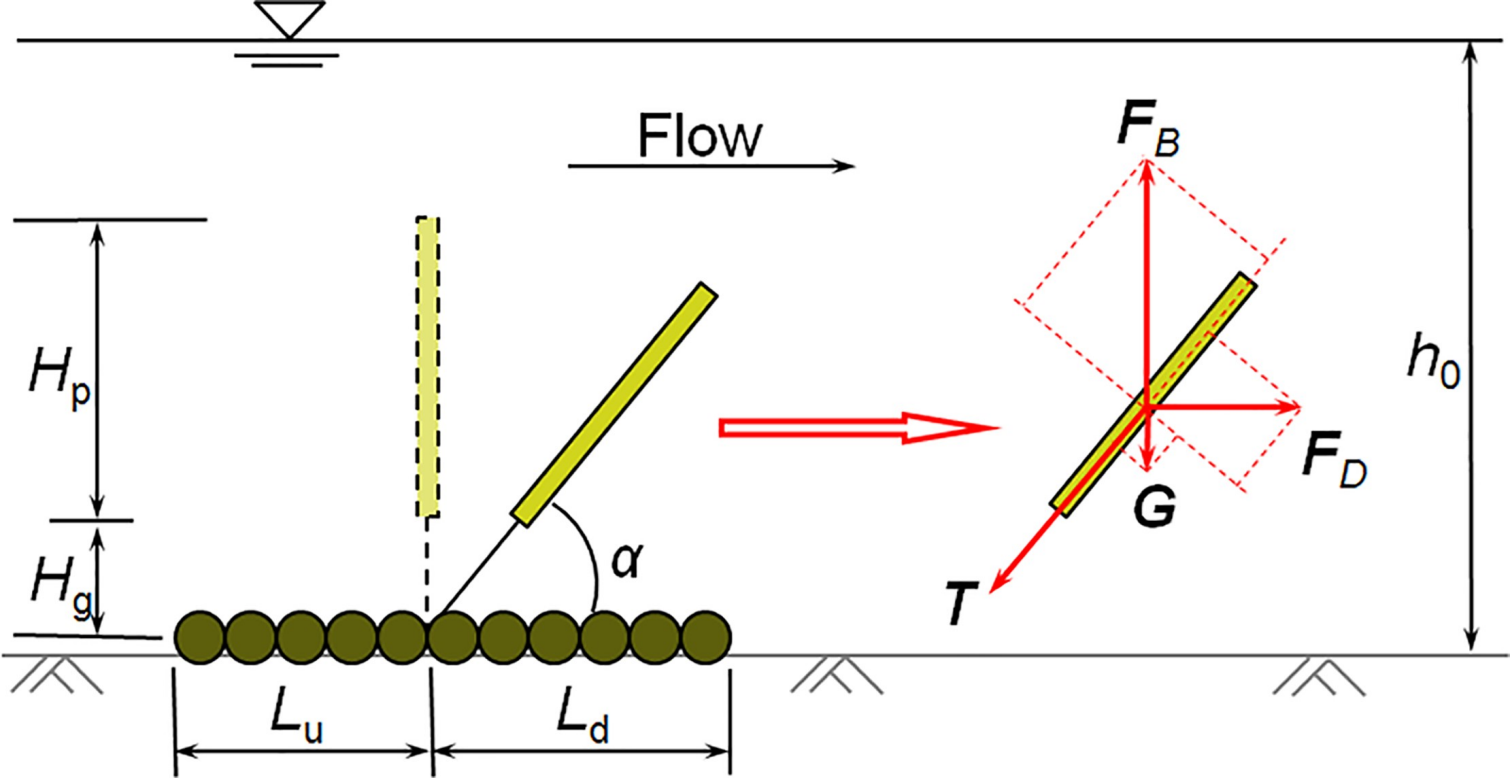
Forces acting on the sloping plate of GMSP. Symbols: *F*_*B*_ = buoyancy force; *F*_*D*_ = flow drag force; *G* = gravity; *T* = tension force; *H*_p_ = height of the sloping plate; *H*_g_ = height of the gap between the plate and the mattress; *L*_u_ = width of geotextile mattress on the upstream side; *L*_d_ = width of geotextile mattress on the downstream side; *h*_0_ = undistributed water depth; *α* = sloping angle of the plate.

At the same time, the geotextile mattress with sloping plate (GMSP) shares the similar features with the GMSC, including the flow patterns and sediment transportation process, for their operation principals are almost the same. However, it should be pointed out that due to the slight difference in structures between the GMSP and the GMSC, the achievements through tests on GMSC may not be applicable to GMSP directly, and vice versa. When the GMSP is placed in steady current, four forces act on the sloping plate (see Fig 7): a drag force (*F*_*D*_) induced by the current, a tension force (*T*) from the string, a buoyancy force (*F*_*B*_) and gravity (*G*). The balance of the four forces is reached when

$$\{\begin{array}{c} G\sin\alpha +T=F_{B}\sin\alpha +F_{D}\cos\alpha \\ G\cos\alpha +F_{D}\sin\alpha =F_{B}\cos\alpha \end{array}$$ (1)

On this occasion, the sloping plate will lean to the downstream side at a steady sloping angle. The sloping plate thus serves as the sloping curtain in the GMSC. The gap between the geotextile mattress and the sloping plate operates as the sand-pass openings. The flow structures are similar to that of the GMSC. Consequently, the problem of seepage failure bothering the GMSC driven by the bed pressure difference is also to appear in GMSP similarly.

## Laboratory model tests

Potential seepage failure under the mattress of a GMSP can be fatal to the GMSP structure, and similar phenomenon has occurred in previous experiments. It is thus important to improve our understanding on the mechanism of the seepage failure. One main aspect of the seepage failure mechanism is the parametric effects of the GMSP on the seepage hydraulic gradient under the mattress, which is a key parameter to predict the onset of seepage failure. The seepage gradient under the mattress is mainly determined by the bed pressure distribution near the GMSP, so the focus of this qualitative mechanism study is the effects of the GMSP parameters on the averaged hydraulic gradient under the mattress. Thus the tests were performed on an unmovable bed. The GMSP models were fixed on the side walls of the flume on both ends and the GMSP parameters were adjusted manually to improve the convenience in the tests.

**Fig 8:**
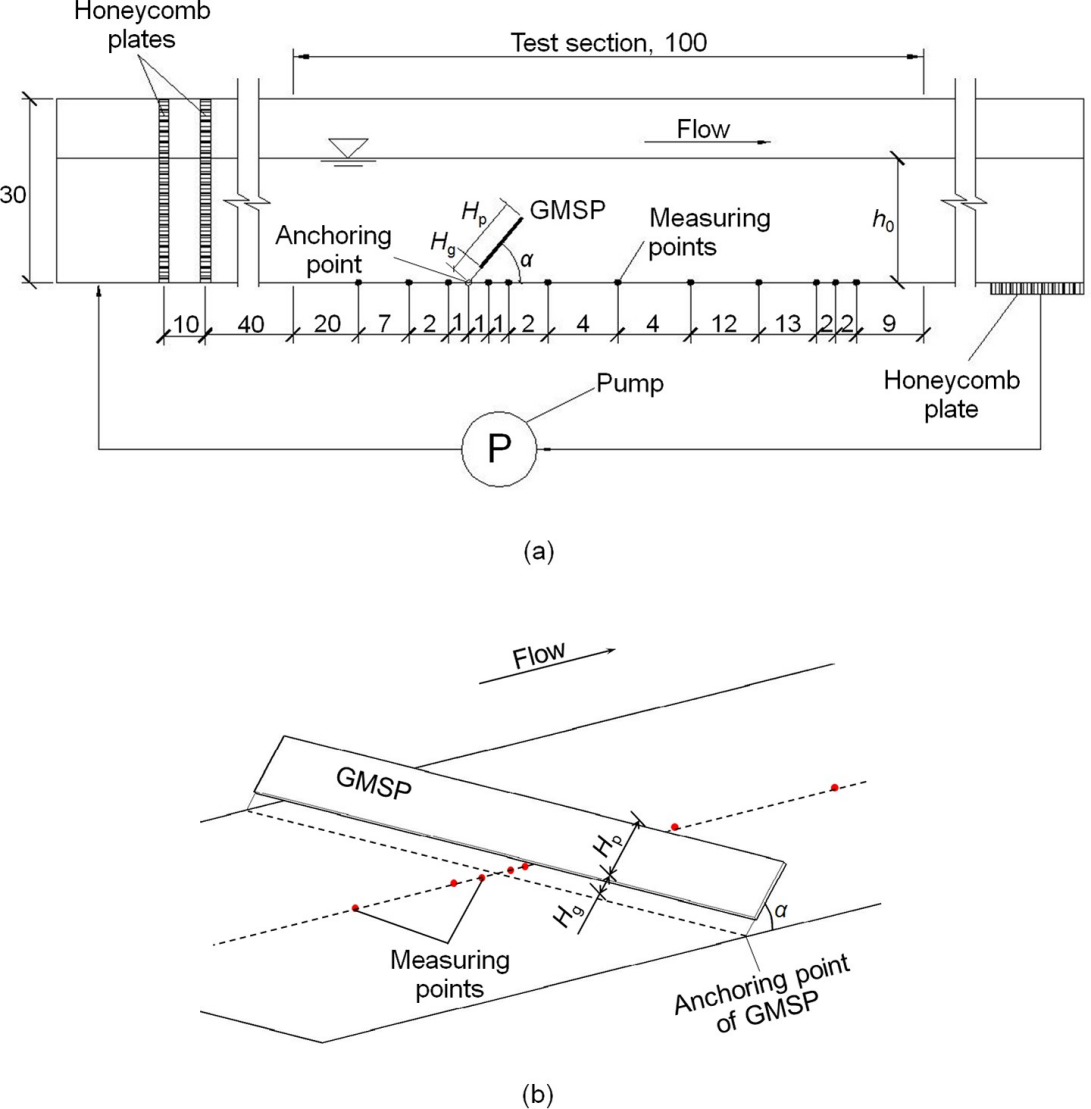
Sketch of test flume. (a) Side view of the experiment flume (Not to scale, Unit: cm). (b) Sketch of the GMSP model in the flume. Symbols: *H*_p_ = height of the sloping plate; *H*_g_ = height of the gap between the plate and the mattress; *α* = sloping angle of the plate; *h*_0_ = undistributed water depth.

The physical experiments were completed in a multi-functional recirculation flume, which was 2.4 m long, 0.3 m wide and 0.35 m deep. The test section, located in the center part of the flume, was 1.0 m long. The side walls and the bottom of the flume were made of Perspex for easy observation of the experiments. A hydraulic pump was used for the current generation, which is capable of generating a steady current up to 0.6 m/s in water depth of 0.1 m. Two honeycomb plates were installed at 0.4 m upstream to the test section with a separation of 0.1 m to stabilize the unidirectional current, as is shown in Fig 8A. Another honeycomb plate was placed downstream to the exit of the test section to minimize the influence of the outflow on the current in the test section. This arrangement ensures the current to be steady within the test section. The experiment setup was designed according to that of Chen and Su [29], who achieved a series of precise flow patterns downstream to a pipeline in steady currents. With the measures taken to ensure accuracy, the results of the experiment can be considered to be convincing for a qualitative mechanism study.

A series of simplified models of the GMSP made of glass were designed for and used in the tests. The width of the GMSP models was 0.3 m, which was the same as that of the flume, and the plate height *H*_p_ varied with cases from 2.5 cm to 4.0 cm. The anchoring point of the model plates was fixed at 0.30 m downstream to the entrance of the test section in all cases (see Fig 8). As this paper aims to reveal the parametric effects of the sloping plate on the bed pressure distribution, the geotextile mattress was omitted in the simplified models to make bed pressure measurement more convenient. The absence of the geotextile mattress could affect the roughness of the bed, increasing the bottom flow velocity in the sand-pass gap [26]. However, in practical engineering projects, the mattress may deform and sink into the bed after installation due to its weight and the top surface of the mattress may be end up at the same level of the bed. In this occasion, the results of clear water flume experiment can be convincing and acceptable for the aim of the present qualitative study is to figure out the mechanism in bed pressure distribution and assess the potential seepage failure under the mattress.

The pressure distribution on the flume bed was measured by 12 water pressure sensors connected to the bed of flume separately. The full range of the pressure sensors was 10 kPa; the accuracy was ±0.001 kPa and the sampling rate was 100 Hz. The digital pressure signals were collected and processed by the pressure sensor and transmitted directly to a personal computer through a concentrator. A SmartSensor software system was used to control the pressure sensors and record the pressure readings. The software system also provides real-time graphical display. All the bed pressure measuring points were located on the centerline of the test section (see Fig 8B). The distance between the entrance of the test section and the measuring points was between 0.20 m and 0.71m, as is shown in Fig 8A. In the experiment, the pressure readings were taken for a period of 3 minutes after the reading became stable to ensure that the readings are independent of time.

To investigate the effects of design parameters of the GMSP, including the sloping angle *α*, the height of the plate *H*_p_ and the opening ratio *δ*, on the pressure distribution on the bed of the flume, a total number of 14 cases were designed for the experiment. The opening ratio of the GMSP *δ* is defined as:

$$\delta =\frac{H_{g}}{H_{p}+H_{g}}$$ (2)

where *H*_g_ is the height of the gap between the sloping plate and the geotextile mattress; *H*_p_ is the height of the sloping plate (see Figs 7 and 8). The water depth *h*_0_ was fixed to be 0.1 m in all cases. The averaged velocity of the current was kept constant as 0.3 m/s. The flow is steady and turbulent in all tests. The Reynolds number based on the height of floating plate *Re*_*H*p_ was between 7500 and 12,000 and the Froude number based on the flow depth was *Fr* = 0.303 for all cases. The cases were divided into three groups. In each group, only one parameter was changed while the others were kept constant. Group A focuses on the sloping angle of GMSP where the sloping angle *α* ranges from 35° to 60°; group B the opening ratio where the opening ratio *δ* is changed between 0.000 and 0.400; group C the plate height of the GMSP where the relative plate height *H*_p_ / *h*_0_ is varied between 0.25 and 0.40. The details of the cases are listed in Table 1.

**Table 1:** Experiment cases.

Group	Case	Sloping angle *α* (°)	Opening ratio *δ*	Relative plate height *H*_p_ / *h*_0_
A	A1	35	0.250	0.30
	A2	40	0.250	0.30
	A3	45	0.250	0.30
	A4	50	0.250	0.30
	A5	55	0.250	0.30
	A6	60	0.250	0.30
B	B1	50	0.000	0.30
	B2	50	0.143	0.30
	B3	50	0.250	0.30
	B4	50	0.333	0.30
	B5	50	0.400	0.30
C	C1	50	0.250	0.25
	C2	50	0.250	0.30
	C3	50	0.250	0.40

## Results and analysis

In this study, the width of the geotextile mattress of the GMSP is selected based on the suggestions of Xie and Liu [23]. The width of mattress on the upstream side (*L*_u_) is 3 cm and the width on the leeside (*L*_d_) is 4 cm (see Fig 7), i.e. the mattress covers a range from *x* = -0.03 m to 0.04 m (*x* = the distance to the anchoring point of the sloping plate, see Fig 8). The averaged seepage hydraulic gradient beneath the mattress *i*_m_ is calculated as:

$$i_{m}=\frac{\Delta p/\rho g}{L_{u}+L_{d}}$$ (3)

where *i*_m_ is the averaged seepage hydraulic gradient beneath the mattress, Δ*p* is the difference of bed pressure readings on two sides of the GMSP (between *x* = -0.03 m and *x* = 0.04 m in this study), *ρ* is the density of water (*ρ* = 1 × 10^3^ kg/m^3^) and *g* is the gravity acceleration (*g* = 9.8 m/s^2^). In this study, *L*_u_ = 0.03 m and *L*_d_ = 0.04 m. In the following parts of this paper, the averaged seepage hydraulic gradient under the mattress *i*_m_ will be used to evaluate the potential of seepage failure under the geotextile mattress.

### Effects of sloping angle

**Fig 9:**
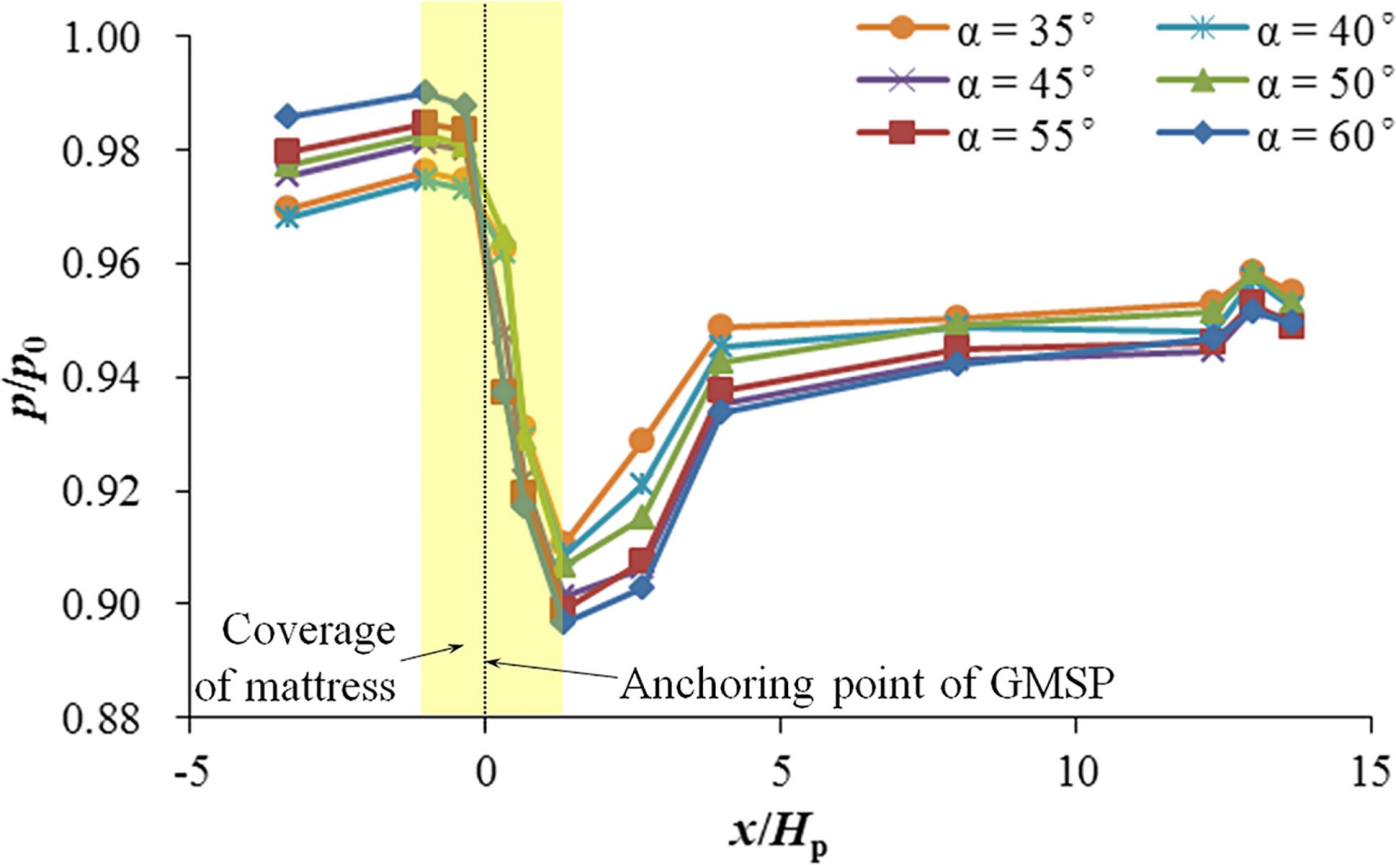
Bed pressure distribution on two sides of the GMSP for different sloping angles.

To investigate the effects of sloping angle on the bed pressure distribution, tests in Group A were designed and performed with sloping angles of the GMSP varying from 35° to 60°. The other two variables were kept constant, i.e. the opening ratio *δ* = 0.250 and the relative plate height *H*_p_ / *h*_0_ = 0.30. Fig 9 shows the bed pressure distribution with different sloping angles. The horizontal axis indicates the normalized distance to the anchoring point of the sloping plate *x* / *H*_p_ and the vertical axis is the non-dimensional pressure *p* / *p*_0_, where *p* is the reading of the pressure gauge and *p*_0_ is the static water pressure on the bed in still water (i.e. *p*_0_ = *ρgh*_0_ = 980 Pa). In Fig 9, all six curves have the similar trend. The pressure drop around the anchoring point of the sloping plate triggered by the GMSP can be clearly observed. Upstream to the pressure drop, a gentle climb in the bed pressure can be witnessed from *x* / *H*_p_ = -3.33 to *x* / *H*_p_ = -1.00 in all six cases. Then the pressure drop begins and hits a nadir at *x* / *H*_p_ = 1.33. The pressure drop can partially be attributed to the fluctuation of water surface profile, which drops by approximately 4 mm from *x* / *H*_p_ = -1.00 to *x* / *H*_p_ = 1.33. The changes in the water surface profile and the flow pattern on the leeside of the GMSP indicate the blockage effect of the model plate [25]. The pressure on the bed downstream to the nadir gradually rises up by approximately 0.037*p*_0_, covering about 48% of the pressure drop on average, but fails to meet the value upstream the GMSP. In addition, the pressure difference shows an increasing trend with the increase of the sloping angle. The pressure difference between the upstream and downstream edges of the mattress increases from 0.065*p*_0_ for *α* = 35° to 0.093*p*_0_ for *α* = 60°.

**Fig 10:**
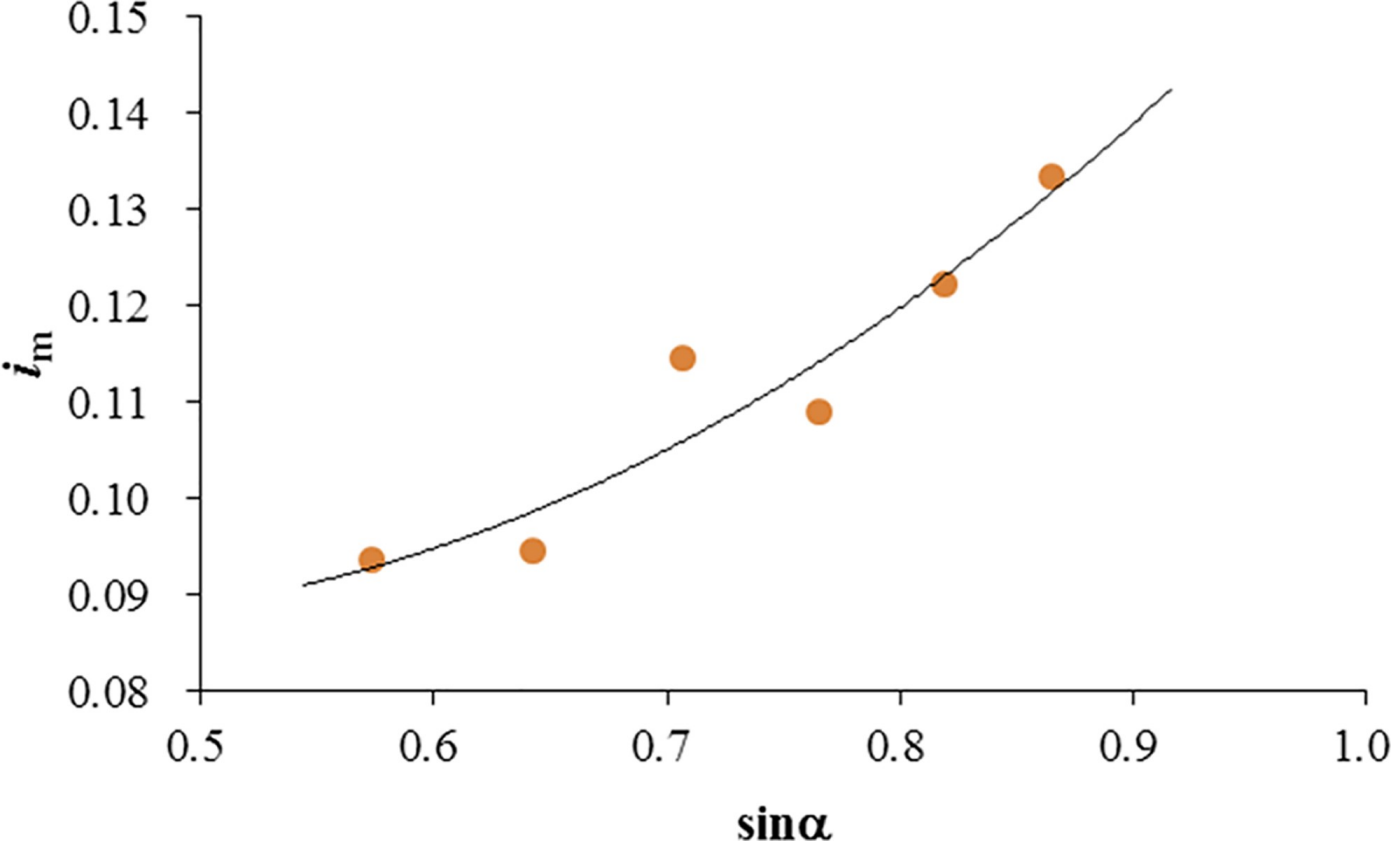
Variation of averaged seepage hydraulic gradient under the GMSP with sloping angle.

Fig 10 shows the variation between the averaged seepage hydraulic gradient beneath the mattress *i*_m_ and the sine value of the GMSP sloping angle (sin*α*), which has a direct proportion with the flow blockage area of the GMSP with the same plate width and height. In general, the averaged seepage hydraulic gradient increases by 42.8% from 0.093 to 0.133 with the increase of sin*α*. This increase of pressure difference may be attributed to the increase of water surface variation due to the increase of flow blockage.

### Effects of the opening ratio

**Fig 11:**
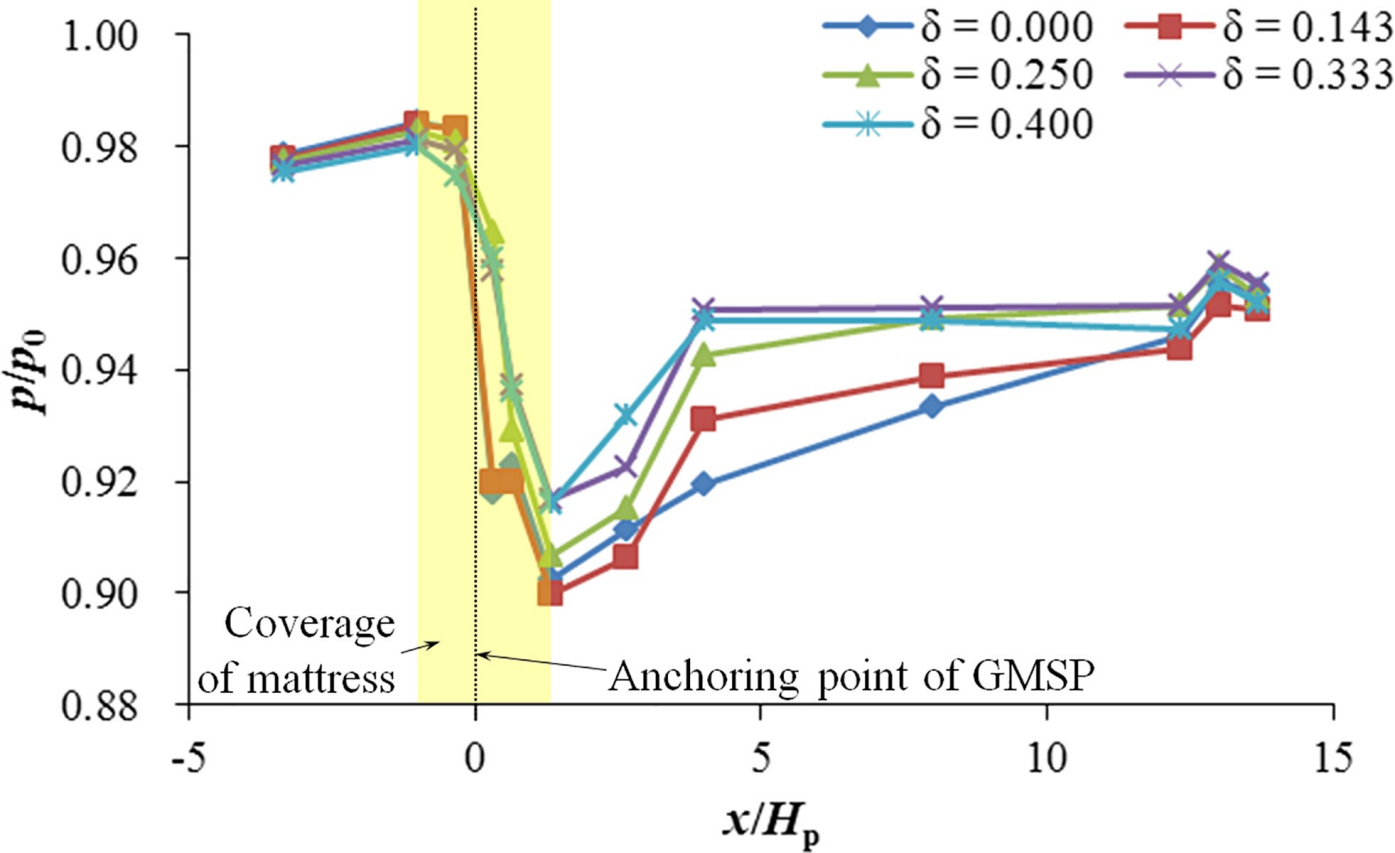
Bed pressure distribution on two sides of the GMSC for different opening ratio.

To study the effects of the opening ratio of the GMSP on the bed pressure distribution, tests in Group B were designed and performed with the opening ratio of the GMSP varying between 0.000 and 0.400. The other two variables were kept constant, i.e. the sloping angle *α* = 50° and the relative plate height *H*_p_ / *h*_0_ = 0.30. Fig 11 shows the bed pressure distribution in different cases for different opening ratios. In Fig 11, similar characteristics can be observed as that in Fig 9. The pressure difference narrows down with the growth of opening ratio *δ* when the *δ* > 0.143. The pressure difference between the upstream and downstream edges of the mattress descends by 23.8% when *δ* increases from 0.143 to 0.400.

**Fig 12:**
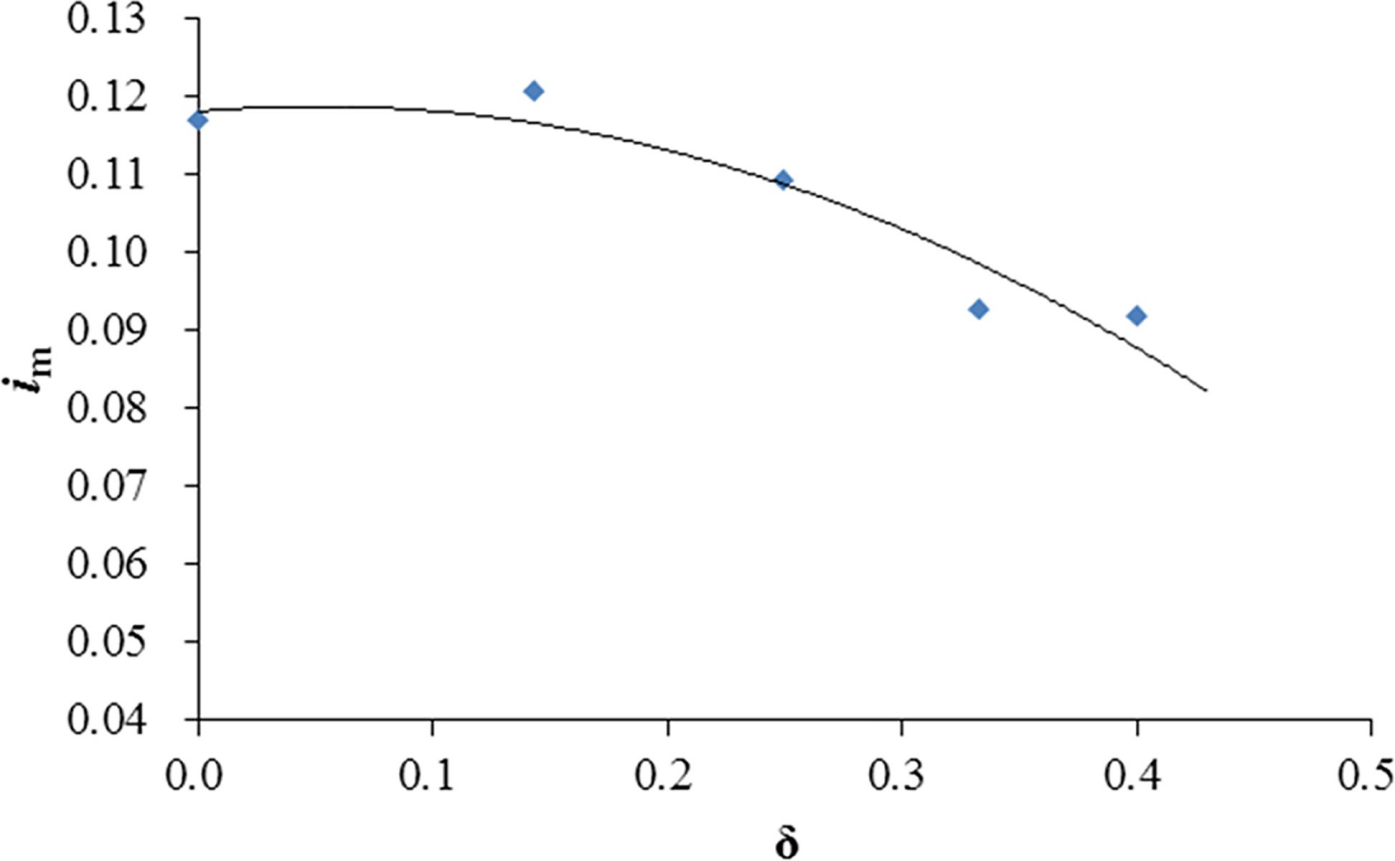
Variation of averaged seepage hydraulic gradient under the GMSP with opening ratio.

Fig 12 shows the relationship between the opening ratio of GMSP and the averaged seepage hydraulic gradient beneath the mattress *i*_m_. The averaged hydraulic gradient slightly increases with the increase of opening ratio when the opening ratio *δ* < 0.143. When the opening ratio is over this value, the averaged hydraulic gradient decreases considerably from *i*_m_ = 0.121 when *δ* = 0.143 to *i*_m_ = 0.092 when *δ* = 0.400. The drop of the hydraulic gradient may involve the fact that the influence of the sloping plate on the bed tends to vanish when the height of the gap between the sloping plate and the mattress increases, i.e. with the rise of the opening ratio.

### Effects of relative plate height

**Fig 13:**
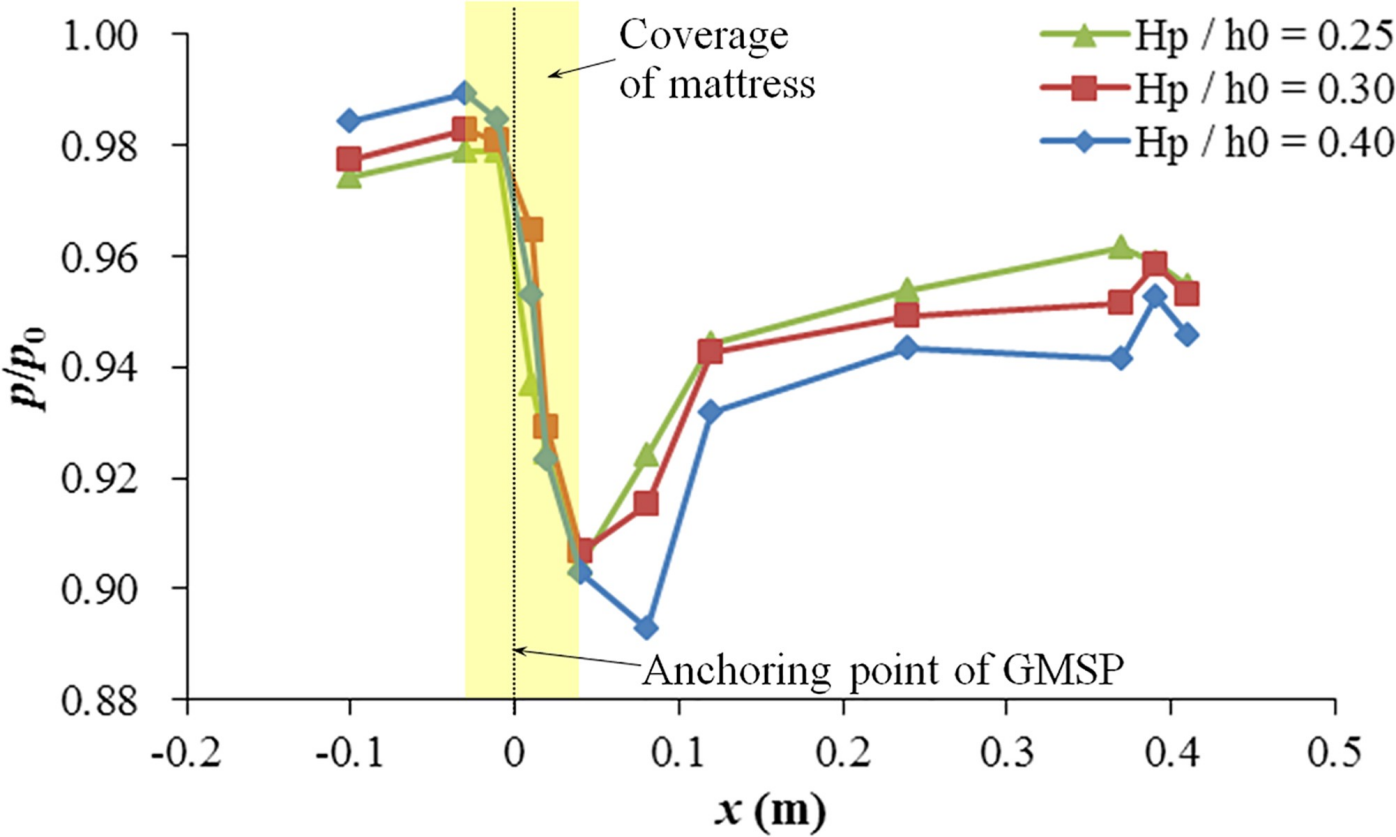
Bed pressure distribution on two sides of the GMSP for different relative plate heights.

To study the effects of the relative plate height of the GMSP on the bed pressure distribution, tests in Group C were designed and conducted with the relative plate height of the GMSP varying between 0.25 and 0.40. The other two variables were kept constant, i.e. the sloping angle *α* = 50° and the opening ratio *δ* = 0.250. Fig 13 shows the bed pressure distribution in different cases with different relative plate height. The basic features of the bed pressure distribution curve are similar to those in Fig 9. However, the lowest point of the pressure distribution curve for *H*_p_ / *h*_0_ = 0.40 (Case 14) locates further downstream than the other two cases (at *x* = 0.08 m, *x* / *H*_p_ = 2.00). This change may be related to the variation in the shape of vortex zones due to the increase of plate height. In addition, considerable increase of pressure difference on two sides of the GMSP can be observed with the increase of relative plate height. The pressure difference between the upstream and downstream edges of the mattress climbs from 0.075*p*_0_ for *H*_p_ / *h*_0_ = 0.25 to 0.086*p*_0_ for *H*_p_ / *h*_0_ = 0.40.

**Fig 14:**
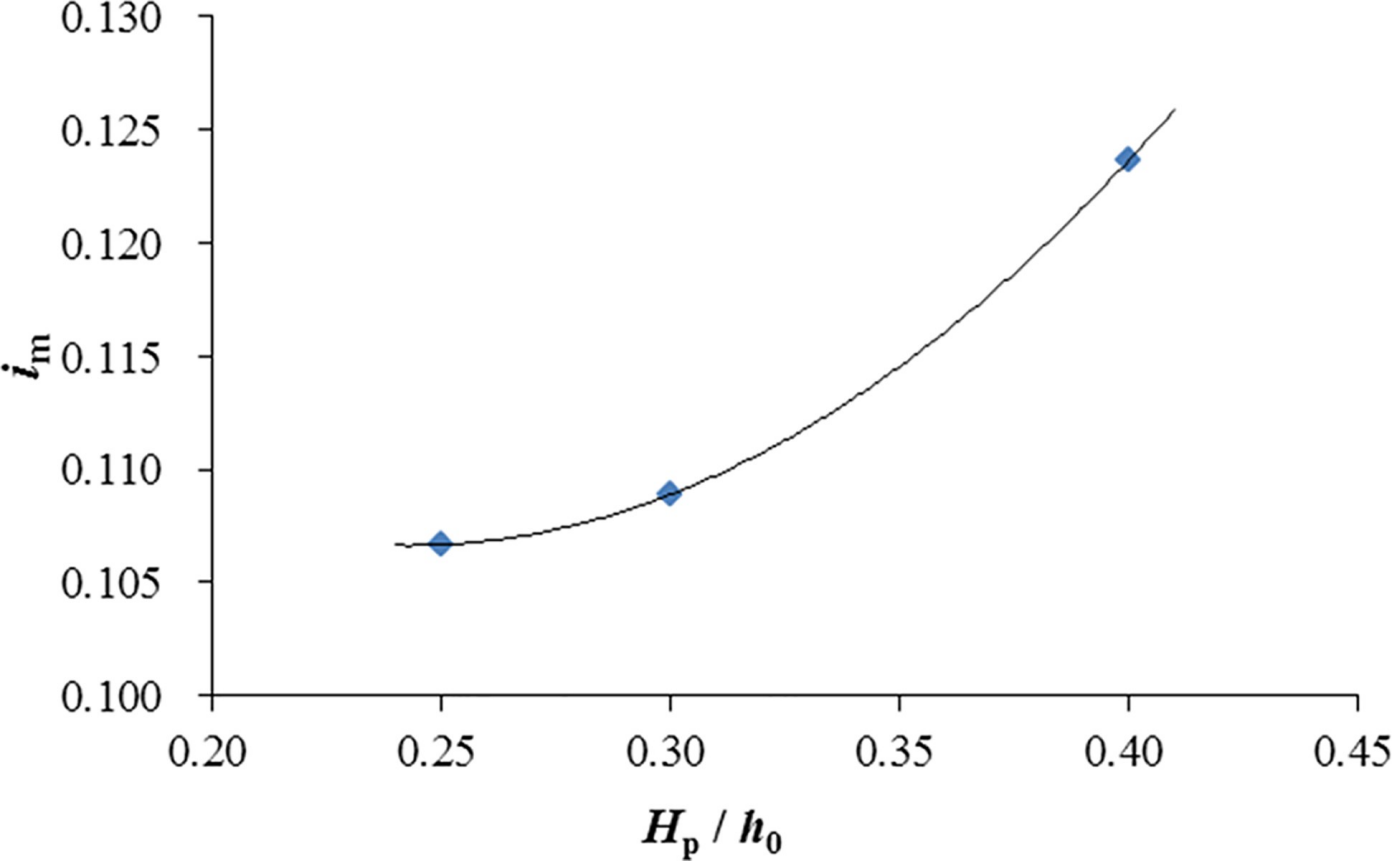
Variation of averaged seepage hydraulic gradient under the GMSP with relative plate height.

Fig 14 shows the relationship between the relative plate height and the averaged seepage hydraulic gradient beneath the mattress *i*_m_. A 16% growth can be seen in the averaged hydraulic gradient with the increase of relative plate height from *H*_p_ / *h*_0_ = 0.25 to 0.40. The increase may also be associated with the increase of flow blockage due to the increase of relative plate height.

### Discussion on the test results

**Fig 15:**
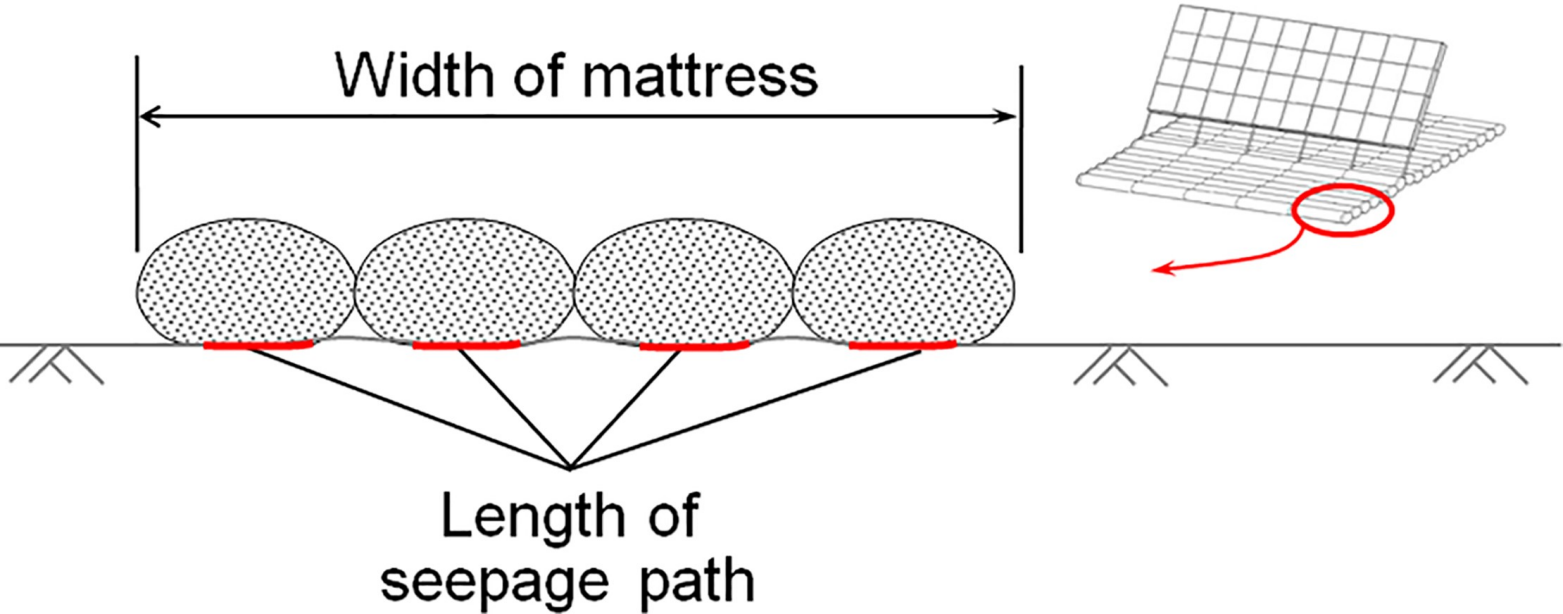
Schematic diagram for the mattress width and the seepage path under the mattress.

**Fig 16:**
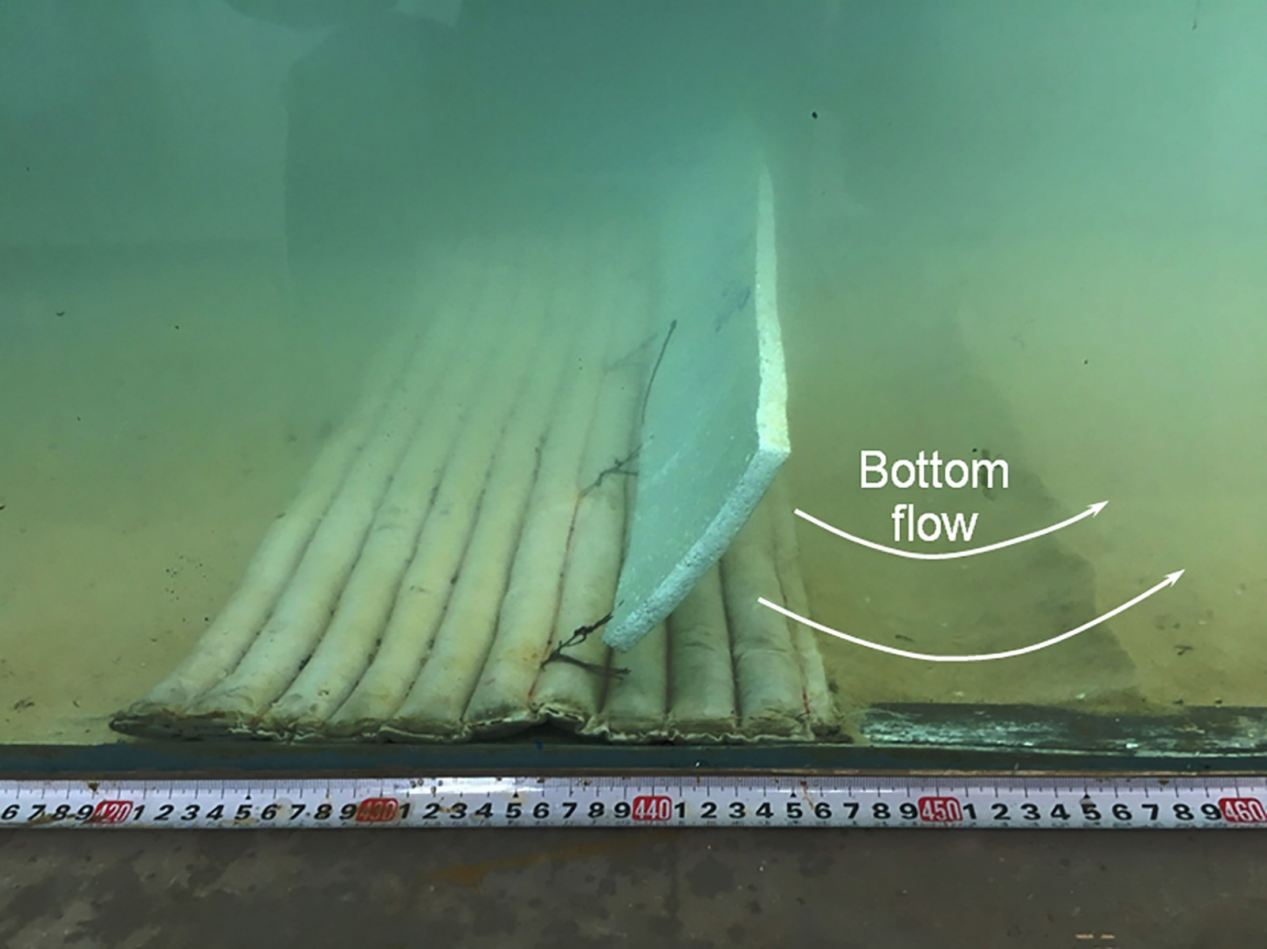
Scour on the leeside edge of the geotextile mattress.

The averaged seepage hydraulic gradient under the mattress *i*_m_ in the aforementioned experiments is much lower than the critical seepage hydraulic gradient of piping which is about 0.9 in some non-cohesion sediments [30]. However, according to the results in a separate live-bed experiment, the critical value of *i*_m_ of the incipient motion of sand particles under the GMSP varied between 0.06 and 0.55 for different mattress widths, opening ratios and plate heights, which is also much smaller than the critical value of piping. This indicates that the safety boundary of the scour under the geotextile mattress cannot be independently determined by the properties of the soil (i.e. the critical hydraulic gradient of piping). Furthermore, the working condition of GMSP in practical engineering projects is far harsher than the setup in the laboratory experiments. The complicated submarine environment will bring uncertainty to the stability of the mattress, so the upper limit of the critical value of *i*_m_ should be further cut down from the critical hydraulic gradient of piping before it is used in the practical engineering project. The effects on the critical value of the averaged hydraulic gradient may be derived from numerous factors and some leading ones are listed below:

(1) Overestimation on seepage path under the geotextile mattress. The geotextile mattress of the GMSP is constituted of a series of oval mattress tubes, as is shown in Fig 6. When the mattress is deployed, not every part on the bottom of the mattress can contact with the bed perfectly (see Fig 15), and some gaps are left between the tubes. The seepage path under the geotextile mattress is actually much shorter than the width of the mattress. The hydraulic gradient under the mattress tubes is thus considerably higher than the averaged hydraulic gradient under the mattress *i*_m_. When a critical point of hydraulic gradient under the mattress tubes is reached, the averaged hydraulic gradient under the mattress *i*_m_ can be well below this value. As a result, the critical value of *i*_m_ gets remarkably smaller than the critical hydraulic gradient of piping.

(2) Slots between the mattress and the sediment surface. Although sand bed was carefully paved where the aforementioned experiment was conducted, some wrinkles may appear on the bottom surfaces of the mattress tubes. The wrinkles provide a perfect place for the slots between the geotextile mattress and the sand bed and thus paths for the flow under the mattress from the upstream side to the leeside. The flow velocity through these paths is much higher than that in seepage for the water head loss is smaller. At a higher velocity, the flow through the slots under the mattress may lead to the scour underneath the mattress even when the value of *i*_m_ does not reach the critical seepage hydraulic gradient of piping.

(3) Scours on the leeside edge of the geotextile mattress. The sand-pass opening below the plate is not only a path for sediment transportation, but it is a source of high velocity bottom flow as well. Xie and Liu [23] detected high velocity flow through the sand-pass opening through numerical simulation. The excessive flow through the opening may extend beyond the mattress on the leeside and trigger scour on the leeside edge of the GMSP. In Fig 16, the scour hole on the leeside of the GMSP can be observed after 20 minutes’ flow. The scour is highly likely to be caused by the high velocity flow through the gap between the sloping plate and the mattress. As the scour hole gets deeper, the barrier slowing down the seepage under the mattress is removed and the critical value of *i*_m_ also gets reduced. Xie et al [24] reckoned that the opening ratio should be strictly controlled below a specific value to prevent the scour of this kind.

(4) Sand waves on sea bed. In practical engineering projects, the GMSP is often installed on a sand bed with sand waves and wrinkles of different scales, instead of a carefully paved sand recess in experiments. After the deployment of geotextile mattress, gaps may appear between the bottom of the mattress and the sand bed. If the gaps are connected, they will become the access for the flow powered by the pressure difference on two sides of the GMSP. The sediment under the mattress can thus be scoured even before the seepage failure occurs.

Therefore, the safety boundary for the averaged seepage hydraulic gradient under the geotextile mattress of the GMSP could get much smaller than the critical hydraulic gradient of piping and it can easily be overwhelmed, especially in practical engineering projects. It is suggested that the parametric design of the GMSP should consider the discontinuous contact between geotextile mattress and seabed and the width of the mattress should be extended to reduce the risk of seepage failure under the GMSP.

## Conclusion

In this study, the Geotextile Mattress with Sloping Plate (GMSP) is proposed based on the simplification of the GMSC with the construction feasibility considered. The bed pressure distribution around the GMSP is measured and the effects of three GMSP parameters on the averaged seepage hydraulic gradient underneath the geotextile mattress of the GMSP are studied. On the basis of the analysis above, the following conclusions can be proposed.

(1) The bed pressure drops remarkably on the downstream side of the GMSP compared with the upstream side. The nadir point of the bed pressure is reached approximately 1.3 times the plate height downstream to the GMSP in most cases. The bed pressure downstream to the nadir point rises but fails to reach the value on the upstream side of the GMSP.

(2) The averaged seepage hydraulic gradient beneath the mattress *i*_m_ increases with *α* increasing from 35° to 60° in general. *i*_m_ also ascends with the increase of *H*_p_ / *h*_0_, but reduces with the increase of opening ratio when the opening ratio *δ* > 0.143.

(3) The safety boundary for the averaged seepage hydraulic gradient under the geotextile mattress of the GMSP could get much smaller than the critical hydraulic gradient of piping and it can easily be overwhelmed, especially in practical engineering projects. The suggestion for the parametric design of the GMSP is to extend the width of mattress to reduce the risk of failure under GMSP.

The findings in this study can be helpful to improve the understanding of the parametric effects of a GMSP on the bed pressure distribution and the seepage hydraulic gradient under the GMSP mattress. Further investigations will focus on the effects on the sloping angle and the forces on the GMSP structure.

## Supporting information

S1 Data Experiment data for all cases. (XLSX)
